# Stress and sleep: impact of the main contributing factors to poor sleep experiences during the COVID-19 pandemic

**DOI:** 10.1192/j.eurpsy.2022.1314

**Published:** 2022-09-01

**Authors:** T. Ionescu, S. Zaharia, E. Minecan, C. Tudose

**Affiliations:** 1 University of Medicine and Pharmacy Carol Davila, Neuroscience 6, Bucharest, Romania; 2 Alexandru Obregia Clinical Hospital of Psychiatry, Child And Adolescent Psychiatry, Bucharest, Romania

**Keywords:** Sleep quality, Insomnia, Covid-19

## Abstract

**Introduction:**

The COVID-19 (Coronavirus Disease 2019) pandemic is associated with several stressful factors that can negatively affect peoples’ sleep quality and mental health.

**Objectives:**

The aim of the current study was to prospectively identify decreased sleep quality and associated risk factors in general population during COVID-19 pandemic.

**Methods:**

We conducted a prospective, observational online study on a Romanian sample of 667 respondents aged >18 years. Sleep quality and quantity was evaluated with Athens Insomnia scale (AIS) and the main concerns associated with the pandemic context were evaluated through a multiple-choice question.

**Results:**

The data collected identified important evidence regarding the prevalence and intensity of insomnia. The average score for AIS was 6.13 (cut-off point for was set at 8). However, it is worth noting that 179 respondents (26.8%) meet the criteria for insomnia. Of the 8 self-assessment items, daytime sleepiness was the criterion evaluated with the highest average score (1.01), all other items getting subunit values. A low quality of sleep was linearly related with fear of illness/death (p=0.053), fear of illness/death of close people (p=0.032), social isolation (p<0.001), economic impact (p=0.003), losing the job (p<0.001) and social stigma associated with COVID-19 infection (p=0.009).

**Conclusions:**

More than a qurter of respondes scored above the threshold of 8 at the insomnia scale, while losing the job, social stigma associated with COVID-19 infection and social isolation are the main risk factors for a low quality and quantity of sleep.

**Disclosure:**

No significant relationships.

